# Treatment-Induced Neuropathy Presenting as Thoracic Radiculopathy and Abdominal Wall Protrusion After Marked Glycemic Improvement With Tirzepatide

**DOI:** 10.7759/cureus.117153

**Published:** 2026-09-29

**Authors:** Kian Bagheri, Erin Buck, Suraj Muley

**Affiliations:** 1 Physical Medicine and Rehabilitation, HonorHealth, Scottsdale, USA; 2 Family Medicine, Naval Hospital Camp Pendleton, Camp Pendleton, USA; 3 Neurology, HonorHealth, Phoenix, USA

**Keywords:** case report, thoracic radiculopathy, tirzepatide glp-1, treatment-induced neuropathy of diabetes (tind), type 2 diabetes mellitus

## Abstract

Treatment-induced neuropathy of diabetes (TIND) is an uncommon acute neuropathic complication associated with marked improvement in glycemic control. It typically presents with severe neuropathic pain and may include autonomic dysfunction, although focal presentations have also been described. We report the case of a 67-year-old man with long-standing type 2 diabetes mellitus who developed severe thoracoabdominal neuropathic pain and abdominal wall protrusion following marked improvement in glycemic control after the initiation of tirzepatide. Hemoglobin A1c decreased from 11.0% to 6.6% over seven months, accompanied by a 17-pound weight loss. Several weeks after this metabolic improvement, he developed burning, stabbing, and electric shock-like thoracoabdominal pain with dysesthesia, hyperesthesia, and abdominal wall weakness. Extensive gastrointestinal, surgical, and structural evaluations were unrevealing. Electrodiagnostic testing demonstrated a generalized sensorimotor polyneuropathy, but needle electromyography of the thoracic or abdominal musculature was not performed. The clinical presentation was consistent with thoracic radiculoneuropathy occurring in the setting of marked glycemic improvement, although TIND could not be definitively established. This case highlights a focal truncal presentation temporally associated with glycemic improvement following tirzepatide therapy and emphasizes the diagnostic challenges in distinguishing TIND from spontaneous diabetic thoracic radiculoneuropathy.

## Introduction

The use of glucagon-like peptide-1 (GLP-1) receptor agonists and dual glucose-dependent insulinotropic polypeptide (GIP)/GLP-1 agonists has increased substantially in recent years for the treatment of type 2 diabetes mellitus and obesity [1,2]. Agents such as tirzepatide can produce substantial reductions in hemoglobin A1c (HbA1c), frequently accompanied by significant weight loss [3,4]. Marked improvement in glycemic control has been associated with treatment-induced neuropathy of diabetes (TIND), a recognized but likely underdiagnosed complication characterized by acute painful neuropathy and, in some patients, autonomic dysfunction [5,6].

Diabetic thoracic radiculoneuropathy is an uncommon manifestation of diabetic neuropathy characterized by acute or subacute neuropathic pain in a unilateral or bilateral thoracoabdominal distribution, often accompanied by sensory abnormalities and, less commonly, motor involvement of the abdominal wall [7,8]. Abdominal wall weakness may produce focal protrusion that can mimic an abdominal hernia or other intra-abdominal pathology, frequently prompting extensive gastrointestinal and surgical evaluation before a neurologic etiology is recognized [9]. Electromyographic abnormalities in the thoracic paraspinal and abdominal wall muscles can support the diagnosis when present [10]. Although TIND classically presents with painful neuropathy, the relationship between marked glycemic improvement and focal thoracic radiculoneuropathy remains incompletely characterized.

## Case presentation

We present a 67-year-old man with long-standing type 2 diabetes mellitus who developed severe thoracoabdominal neuropathic pain and abdominal wall protrusion following marked improvement in glycemic control after the initiation of tirzepatide (Figure 1). The presentation raised concern for TIND with a focal thoracic radiculoneuropathic phenotype, although the available clinical and electrodiagnostic data did not permit definitive confirmation.

**Figure 1:**
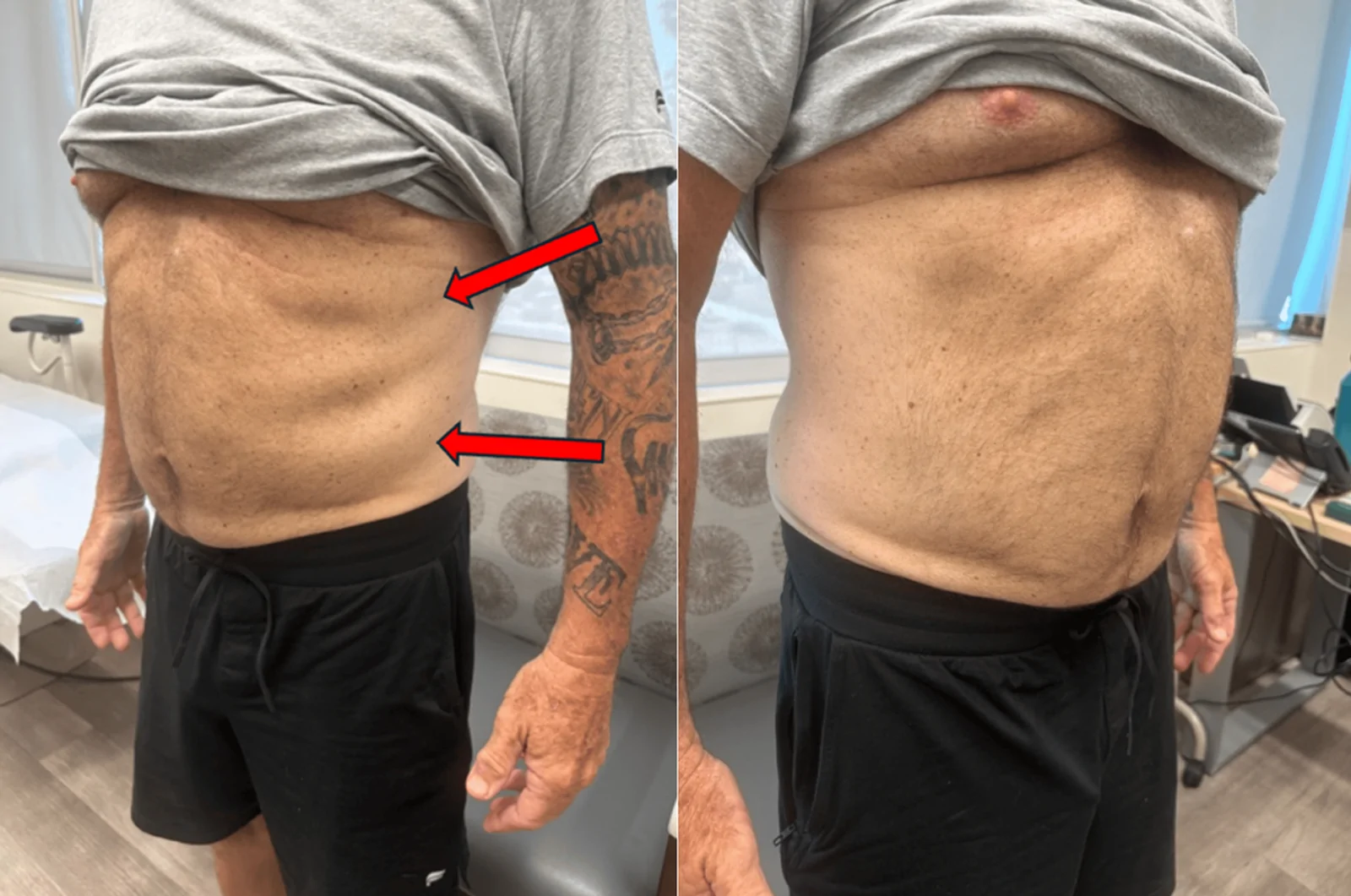
Comparison of abdominal wall appearance showing left-sided abdominal wall protuberance and relative laxity compared to the right side (red arrows), consistent with suspected segmental abdominal wall denervation in the setting of thoracic radiculopathy.

The patient had a 20-year history of type 2 diabetes mellitus complicated by mild distal symmetric polyneuropathy. His baseline HbA1c was 11.0%. In preparation for elective orthopedic surgery, tirzepatide was initiated, followed by substantial improvement in glycemic control. Over approximately seven months, HbA1c decreased to 6.6%, accompanied by a 17-pound weight loss. Intermediate HbA1c or blood glucose measurements were not available.

Several weeks after this metabolic improvement, the patient initially developed bilateral flank discomfort that subsequently evolved into severe, persistent left-sided thoracoabdominal pain. The pain was described as burning, stabbing, and electric shock-like and was accompanied by marked dysesthesia and cutaneous hyperesthesia. He subsequently noted a sensation of left abdominal wall weakness with visible abdominal wall protrusion. A quantitative pain score was not available.

Initial evaluation included abdominal computed tomography (CT), abdominal ultrasound, and gastrointestinal evaluation, which did not identify an intra-abdominal or structural cause for the symptoms. Review of abdominal imaging demonstrated no true ventral abdominal wall hernia or fascial defect. Asymmetric thinning of the left rectus abdominis musculature compared with the contralateral side was reported. Exact measurements and the precise level of maximal asymmetry were not available in the records reviewed. The imaging appearance was considered consistent with, but not diagnostic of, possible segmental abdominal wall denervation.

Empiric treatment for presumed infectious or gastrointestinal etiologies, including treatment directed toward *Helicobacter pylori*, did not provide benefit. Thoracic spine magnetic resonance imaging (MRI) demonstrated no compressive lesion, and surgical spine consultation did not identify a structural cause.

Neurologic examination demonstrated allodynia in the left thoracoabdominal region and subtle weakness of the left abdominal wall musculature. The available examination documentation did not include formal manual muscle testing of the abdominal wall or a detailed dermatomal sensory map. The clinical findings raised concern for thoracic radiculoneuropathy with segmental motor involvement.

Nerve conduction studies demonstrated a generalized sensorimotor polyneuropathy. Sensory studies showed reduced amplitudes in the right median, ulnar, radial, and bilateral sural nerves, with prolonged peak latencies in the median and ulnar nerves and reduced conduction velocities in the radial and sural nerves. Motor studies demonstrated slowed conduction velocities involving the right median nerve (42 m/s), right ulnar nerve (44 m/s across the forearm and 34 m/s across the elbow segment), bilateral peroneal nerves (34-35 m/s), and bilateral tibial nerves (31-35 m/s). Distal motor amplitudes were relatively preserved. Bilateral tibial H-reflexes were not recordable. No pre-treatment electrodiagnostic study was available for comparison. Needle electromyography of the thoracic paraspinal or abdominal wall musculature was not performed; therefore, the study could not directly confirm segmental thoracic radiculopathy or abdominal wall denervation. The electrodiagnostic findings were interpreted in the context of the patient's known diabetes as a generalized sensorimotor polyneuropathy.

The patient was treated with gabapentin, titrated to 300 mg three times daily with planned escalation to 600 mg three times daily, without significant improvement at the time of evaluation. He was not receiving active antihyperglycemic therapy at presentation because of his recent improvement in glycemic control. Given the severity and focal nature of the symptoms and consideration of an inflammatory or immune-mediated radiculoneuropathic process in the differential diagnosis, a trial of high-dose corticosteroid therapy with prednisone 250 mg once weekly for six weeks was subsequently initiated. This represented a pulsed rather than daily corticosteroid regimen. Corticosteroid therapy is not an established standard treatment for TIND, and potential adverse effects, including worsening glycemic control, were considered. Clinical response to corticosteroid therapy was not available at the time of manuscript preparation.

The patient's clinical course is summarized in Table 1.

**Table 1:** Timeline of the clinical course. MRI: magnetic resonance imaging; CT: computed tomography; HbA1c: hemoglobin A1c

Timepoint	Key event
Baseline	Long-standing type 2 diabetes mellitus for approximately 20 years; HbA1c 11.0%
Month 0	Tirzepatide initiated in preparation for elective orthopedic surgery
Month 7	HbA1c decreased to 6.6%; approximately 17-pound weight loss documented
Several weeks after glycemic improvement	Initial bilateral flank discomfort developed
Subsequent weeks	Symptoms evolved into severe left-sided thoracoabdominal neuropathic pain with dysesthesia and hyperesthesia
Later course	Left abdominal wall weakness and visible protrusion developed
Diagnostic evaluation	CT of the abdomen/pelvis, abdominal ultrasound, gastrointestinal evaluation, and thoracic spine MRI did not identify a structural or visceral cause
Electrodiagnostic evaluation	Generalized sensorimotor polyneuropathy identified; bilateral tibial H-reflexes were not recordable. No needle electromyography of thoracic or abdominal musculature was performed
Treatment	Gabapentin provided no significant improvement. Prednisone 250 mg once weekly for six weeks was subsequently initiated; treatment response was not available at the time of manuscript preparation

## Discussion

This case describes severe thoracoabdominal neuropathic pain and abdominal wall protrusion occurring after marked improvement in glycemic control following the initiation of tirzepatide. The temporal association raised concern for TIND with focal thoracic radiculoneuropathic involvement. However, the available data do not establish TIND as the definitive diagnosis, and spontaneous diabetic thoracic radiculoneuropathy remains an important alternative explanation.

TIND is a recognized complication of substantial improvement in glycemic control, particularly in individuals with longstanding poorly controlled diabetes [5,6]. The proposed diagnosis is generally supported by the temporal relationship between a substantial decline in HbA1c and the subsequent development of acute neuropathic symptoms. In this case, HbA1c decreased from 11.0% to 6.6% over approximately seven months. However, intermediate HbA1c values were unavailable, and therefore, the rate of glycemic decline over shorter intervals could not be established. Similarly, the exact interval between the glycemic change and symptom onset was not documented beyond several weeks. Consequently, the available data do not permit confirmation that conventional temporal and glycemic criteria for TIND were met.

The patient also experienced a 17-pound weight loss during the seven-month period of glycemic improvement. Nutritional or micronutrient abnormalities were not assessed, including vitamin B12, thiamine, copper, and vitamin B6 levels. Although the available history does not establish a nutritional deficiency, these possibilities cannot be definitively excluded and represent additional limitations when attributing the neuropathic syndrome solely to glycemic improvement.

Electrodiagnostic testing demonstrated a generalized sensorimotor polyneuropathy, consistent with the patient's known diabetic neuropathy. These findings document an underlying diffuse neuropathic process but do not independently establish the presence of thoracic radiculopathy. In particular, there was no pre-treatment electrodiagnostic study to determine whether the generalized abnormalities represented a change from baseline. In addition, needle electromyography of the thoracic paraspinal or abdominal wall musculature was not performed. The bilateral non-recordable tibial H-reflexes further support peripheral nerve involvement but do not localize the patient's focal thoracoabdominal symptoms to the thoracic roots. Autonomic testing was also not performed.

Diabetic thoracic radiculoneuropathy is a recognized focal complication of diabetes that can present with severe thoracoabdominal neuropathic pain and, in some cases, abdominal wall weakness or pseudohernia [7-9]. The absence of a true fascial defect and the presence of asymmetric rectus abdominis thinning in this case are compatible with this clinical phenotype. However, negative structural imaging should be interpreted primarily as reducing the likelihood of alternative structural causes rather than as positive evidence for TIND. The absence of confirmatory needle electromyography also limits the ability to establish segmental denervation.

An important alternative diagnosis is spontaneous diabetic thoracic radiculoneuropathy occurring independently of TIND. This condition can occur in patients with longstanding diabetes and may produce a clinical presentation very similar to that observed in this patient, including severe thoracoabdominal pain and abdominal wall weakness [11]. The patient's longstanding diabetes, established distal polyneuropathy, and lack of pre-treatment electrodiagnostic testing make it impossible to determine whether the thoracic syndrome represented a manifestation of chronic diabetic neuropathy, a spontaneous diabetic radiculoneuropathy, TIND, or an overlap of these processes. The temporal relationship with marked glycemic improvement raises clinical suspicion for TIND but does not establish causality [12,13].

The use of corticosteroids in this case also warrants consideration. Prednisone was selected because an inflammatory or immune-mediated radiculoneuropathic process was considered given the severity and focal nature of the symptoms. However, corticosteroid therapy is not an established standard treatment for TIND, and there is insufficient evidence from this case to determine whether corticosteroid therapy provided benefit. Furthermore, corticosteroids may adversely affect glycemic control, which is particularly relevant in patients being evaluated for neuropathy temporally associated with glycemic changes. Because treatment response was not available at the time of manuscript preparation, no conclusion regarding corticosteroid efficacy can be drawn.

The clinical presentation also has potential implications for rehabilitation and functional management. Abdominal wall weakness can interfere with trunk stability and may contribute to functional limitations even when structural abdominal pathology is absent. Management should therefore include monitoring of abdominal wall strength and function, appropriate neuropathic pain treatment, and consideration of rehabilitation interventions when weakness persists. In patients with suspected diabetic thoracic radiculoneuropathy, physical examination and electrodiagnostic localization may help guide rehabilitation planning and distinguish neurologic weakness from structural abdominal pathology.

The principal contribution of this case is the description of a focal thoracoabdominal syndrome with abdominal wall protrusion occurring in temporal association with marked glycemic improvement after the initiation of tirzepatide. Rather than establishing a new diagnostic phenotype of TIND, the case illustrates the diagnostic overlap between TIND and spontaneous diabetic thoracic radiculoneuropathy and highlights the need for careful temporal assessment and electrodiagnostic localization when focal truncal symptoms occur after substantial improvement in glycemic control.

## Conclusions

Marked improvement in glycemic control was temporally associated with the development of severe thoracoabdominal neuropathic pain and abdominal wall protrusion in a patient with longstanding type 2 diabetes following the initiation of tirzepatide. The clinical presentation was consistent with thoracic radiculoneuropathy and raised concern for TIND, but the available data do not establish a definitive causal relationship. In particular, intermediate HbA1c measurements, precise symptom-onset timing, pre-treatment electrodiagnostic testing, thoracic needle electromyography, autonomic testing, quantitative pain assessments, and treatment-response data were unavailable.

The case highlights the diagnostic overlap between TIND and spontaneous diabetic thoracic radiculoneuropathy. Clinicians should consider neurologic causes when patients with diabetes develop unexplained thoracoabdominal pain or abdominal wall protrusion after substantial improvement in glycemic control while recognizing that temporal association alone does not establish TIND. Early recognition may help avoid unnecessary evaluation for gastrointestinal or structural abdominal pathology and facilitate appropriate neuropathic pain and rehabilitative management.
